# Effective Elastic Behavior of Irregular Closed-Cell Foams

**DOI:** 10.3390/ma11112100

**Published:** 2018-10-25

**Authors:** Wenqi Zhu, Nawfal Blal, Salvatore Cunsolo, Dominique Baillis, Paul-Marie Michaud

**Affiliations:** 1 Univ Lyon, INSA-Lyon, CNRS UMR5259, LaMCoS, F-69621 Villeurbanne, France; wenqi.zhu@insa-lyon.fr (W.Z.); sal.cuns@gmail.com (S.C.); dominique.baillis@insa-lyon.fr (D.B.);; 2 Zhejiang University, Zhejiang-California International Nanosystems Institute, Hangzhou 310058, China; 3 EC2-MODELISATION, 66 Boulevard Niels Bohr, F-69603 Villeurbanne, France; paul-marie.michaud@ec2-modelisation.fr

**Keywords:** irregular closed-cell foams, tomography, periodic computational homogenization, mechanical properties

## Abstract

This paper focuses on the computational modeling of the effective elastic properties of irregular closed-cell foams. The recent Hill’s lemma periodic computational homogenization approach is used to predict the effective elastic properties. Three-dimensional (3D) rendering is reconstructed with the tomography slices of the real irregular closed-cell foam. Its morphological description is analysed to generate realistic numerical closed-cell structures by the Voronoi-based approach. The influences of the Representative Volume Element (RVE) parameters (i.e., the number of realizations and the volume of RVE) and the relative density on the effective elastic properties are studied. Special emphasis is placed on the appropriate choice of boundary conditions. Satisfying agreements between the homogenized results and the experimental results are observed.

## 1. Introduction

In recent years, cellular media such as plastic, ceramic, and metal foams have attracted more and more attention thanks to their interesting mechanical properties [1,2,3,4,5] and thermal properties [6,7,8,9,10].

Concerning the elastic behavior of closed-cell foams, different isotropic random closed-cell foam models based on Voronoi tessellations and level-cut Gaussian random fields have been generated [11]. The density and microstructure dependence of the Young’s modulus and Poisson’s ratio have been computed. The results can be described by the power law $E\propto \rho^{n}\left(1<n<2\right)$ for the closed-cell cellular materials. The microstructure for the RVE of solid foams was determined randomly using a Voronoi tessellation in Laguerre geometry, and the homogenization scheme was applied to assess the effective material response [12]. The influences of the cell wall thickness and cell size variation on the stiffness of closed-cell structure foams were investigated in [13] with Laguerre tessellation models. It was reported that the stiffness of closed-cell cellular solids is insensitive to the variation in wall thickness and the elastic moduli decrease with the increase in the cell size variation. The influence of the internal pore distribution on the elastic properties of closed-cell aluminium foam was investigated by conducting a monotonic compression test in [14]. The effects of the relative density and distribution of solids between the edges and walls on the elastic behaviors of isotropic closed-cell foams were reported in [15] with stochastic equilibrium finite element foam models. Expansion of the analytic coupling functions was proposed to estimate the elastic behaviors of foam materials accurately. Numerical calculations of the relative Young’s modulus on computer-generated Kelvin and random microstructures of closed-cell cellular materials were carried out in [16]. The numerical results were compared to several analytical models.

X-ray tomography was verified as a powerful, non-destructive approach which can provide the images of heterogeneous materials at the relevant scale directly [17]. Tomography technique was used to describe the microstructures of foam materials and realistic foam models were generated with the obtained morphological description [18,19,20,21]. The structures directly reconstructed with tomography slices are usually non-periodic; thus, it is difficult to impose the periodic boundary condition on non-periodic structures [21]. While the Voronoi diagram [22] is usually used to generate periodic foam structures [10,21,23,24,25]. In this current work, we investigate the influences of different boundary conditions on the results.

Numerous numerical homogenization approaches [26,27,28,29,30,31,32,33] have been presented to solve the problems of modeling and calculating the effective behavior of heterogeneous materials. Yu and Tang [34] proposed a method named the variational asymptotic method for unit cell homogenization (VAMUCH) in which a variational statement is formulated with an asymptotic expansion of the energy functional. Inspired by this VAMUCH method, Zhu et al. [25] recently proposed a micromechanical modeling approach based on Hill’s lemma to determine the effective elastic properties of open-cell foams. These two approaches have the advantages that with only one finite element computation, one can obtain the effective properties without imposing any specific boundary loadings or averaging the local fields.

In this study, we are interested in polymer closed-cell foams with light density, which have been investigated widely in regard to their thermal properties, while there have been few studies on the elastic behavior. The tomography slices of the real irregular closed-cell foam are analysed to obtain the dispersion of cell size distribution and to verify the isotropic behavior. Numerical irregular closed-cell structures are generated by the recent Voronoi-based approach using morphological descriptions [35]. Hill’s lemma periodic computational homogenization approach [21,25] is used to predict the effective elastic properties of irregular closed-cell foam models. The influence of the Representative Volume Element (RVE) parameters (the number of realizations and the RVE volume) and the relative density are analyzed. The homogenized results are compared with the results of the tomography reconstruction model and the experimental results.

## 2. Modeling of Irregular Closed-Cell Structure

### 2.1. Material and Its Properties

**Figure 1:**
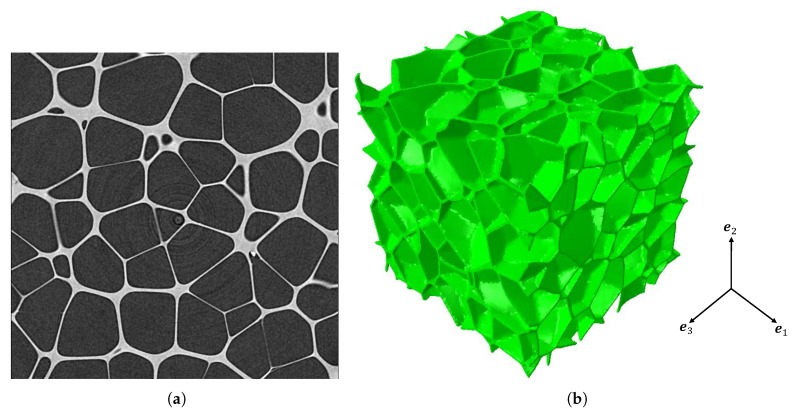
Tomographic data of the irregular closed-cell structure: (**a**) a tomography slice (400 × 400 px${}^{2}$); (**b**) 3D rendering after reconstruction (400 × 400 × 400 px${}^{3}$).

The material used in this study was a modified polyvinylchloride (PVC) cross-linked cellular foam made by AIREX${}^{®}$ (Alcan Airex AG, CH-5643 Sins, Switzerland). The nominal density was 200 kg/m${}^{3}$, named C70.200. A typical tomography slice of the irregular closed-cell foam and the structure obtained by micro-computed tomography are shown in Figure 1. The resolution of the sample was 3 μm per voxel.

Due to different production processes, the properties of PVC can be very different. Ashby pointed out that the Young’s modulus of PVC is 2140–4140 MPa and the density is 1300–1580 kg/m${}^{3}$ (equivalently, the relative density is 12.66–15.38%) [36]. In the current study, the relative density of the foam was set as 15% first, and its influence on the effective elastic properties was estimated in the certain range, as detailed in Section 4.2. The Young’s modulus and the Poisson’s ratio of PVC were set as 4000 MPa and 0.33, respectively [24,36].

### 2.2. Morphological Description

Three morphological parameters were considered when generating realistic numerical irregular closed-cell structures:

1. The relative density $\rho^{\mathrm{hom}}/\rho^{b}$ (in this paper, the superscript notations ${}^{\mathrm{hom}}$ and ${}^{b}$ stand for homogenized property and bulk property, respectively), which was introduced in the previous section;
2. The coefficient of variation $C_{V}$, which represents the dispersion of cell size distribution;
3. The anisotropy of the structure.

The last two parameters are introduced in detail in the following sections.

#### 2.2.1. Dispersion of Cell Size Distribution

The coefficient of variation $C_{V}$ describes the dispersion of cell size distribution with $C_{V}=\sigma_{d_{c}}/\bar{d_{c}}$, where $\sigma_{d_{c}}$ represents the standard deviation of the cell diameters, and $\bar{d_{c}}$ is the average cell diameter (equivalent sphere) [21,25]. The volume of each cell can be measured directly by the software iMorph (Aix-Marseille Université, Marseille, France) [37]. Considering the cells equivalent to the spheres, the distribution of the normalized cell diameters (by the average diameter) exhibits a Gaussian unimodal shape (shown in Section 2.3). By analysing $\sigma_{d_{c}}$ and $\bar{d_{c}}$, the coefficient of variation $C_{V}$ can be estimated to be 0.136. It should be noted that the analysis of the dispersion of cell size distribution was performed on fully contained cells.

#### 2.2.2. Anisotropy

**Figure 2:**
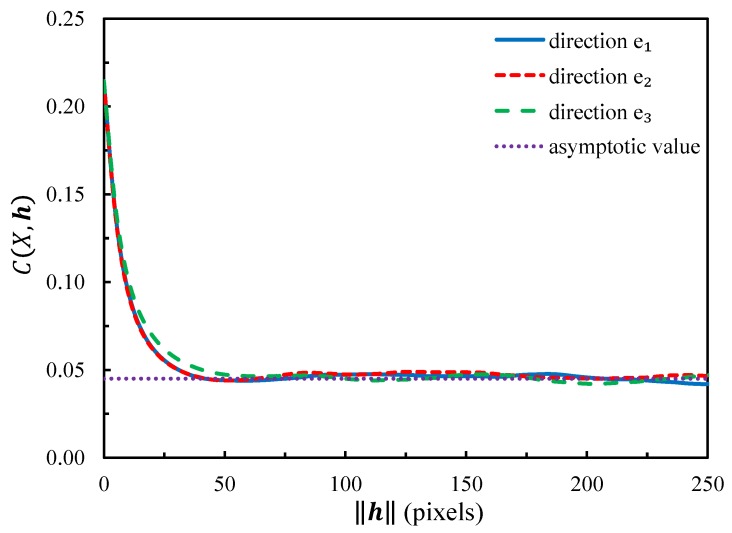
Covariance functions in three directions of the solid phase of closed-cell sample microstructure.

Due to performance needs or manufacturing processes, foams can sometimes exhibit anisotropic behavior. The covariance function is usually used to verify if the microstructure is isotropic or not with the tomography slices [21,38,39]. Figure 2 shows the covariance of the microstructure of the irregular closed-cell foam in the orthogonal coordinate system ($e_{1}$, $e_{2}$, $e_{3}$). In the figure, the microstructure seems to be isotropic, since the covariance functions are similar along three directions.

Another method was used to verify the isotropy, i.e., analysing the matrix of inertia [40]. Cells of the tomography structure were considered to be equivalent to ellipsoids, and the anisotropy of the whole structure was quantified directly by the commercial FE package Abaqus 6.11-2 (Dassault Systèmes Simulia Corp., Providence, RI, USA) [41]. For simplicity, the matrix of inertia was normalized by the first component of the main diagonal, i.e.,

(1) $$\left[I_{C}^{*}\right]=\left[\begin{array}{ccc} 1.00 & ∼0 & ∼0\\ ∼0 & 1.01 & ∼0\\ ∼0 & ∼0 & 1.00 \end{array}\right].$$

The eigenvalues of this matrix are approximately equal.

Hence, the tomography structure is considered to be geometrically isotropic by both methods.

### 2.3. Generation of Numerical Models

**Figure 3:**
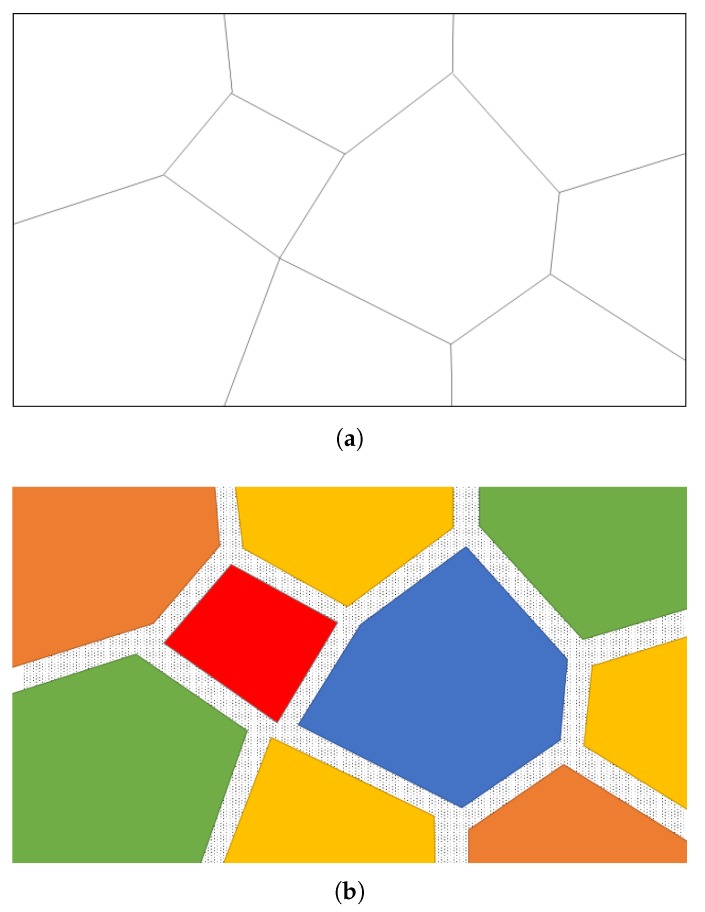
Original (**a**) and exploded (**b**) cell structure. The colors identify cells. The walls are pattern filled.

**Figure 4:**
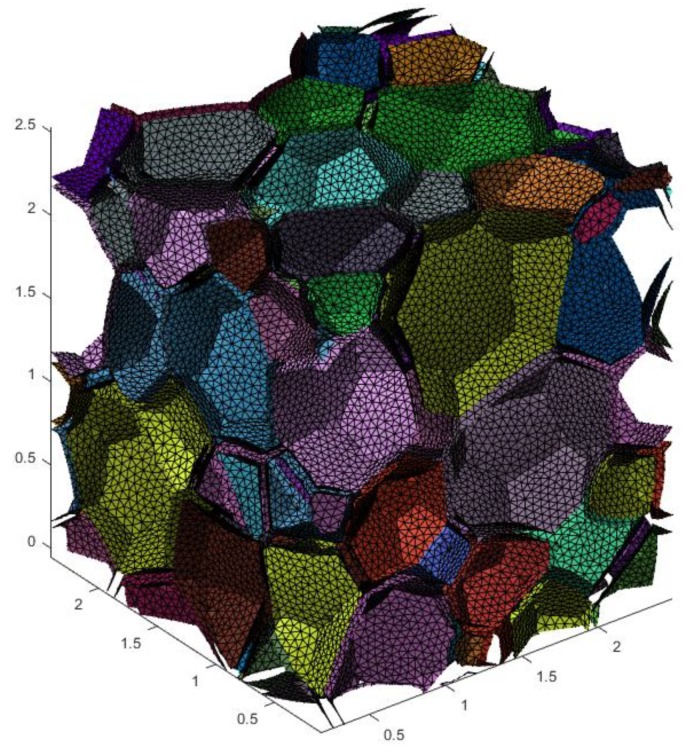
Full cell structure showing the spaces between cells (wall thickness) in 3D.

The recent Voronoi-based approach [25,35], which has been used for open-cell structures before, was extended to numerically generate periodic irregular closed-cell structures. The general generation process can be found in the above papers. Various software, i.e., VORO++, Surface Evolver [42] and Matlab [43], can be used to support the generation and treatment of periodic structures [21]. Surface Evolver helps to generate an energetically stable structure rather than just a random one, which makes the resulting structure more realistic. Moreover, the cell size distribution can be directly specified and finely controlled in Surface Evolver. For closed-cell structures, the cells obtained by Surface Evolver will be “separated”, i.e., every face is duplicated and each of the two copies is grouped with other connected faces, with each group constituting the boundaries of one of the original cells. Each cell-group can then be assigned with a displacement vector. One can see in Figure 3 that with an opportunely chosen set of displacement vectors, a wall with the chosen thickness between cells can be represented. The role of displacement vectors for the full cell structure can be observed in Figure 4. After that, the displaced cells can be used to carve holes in a cubic bounding box, thus resulting in the finalized constant thickness closed-cell structure.

**Figure 5:**
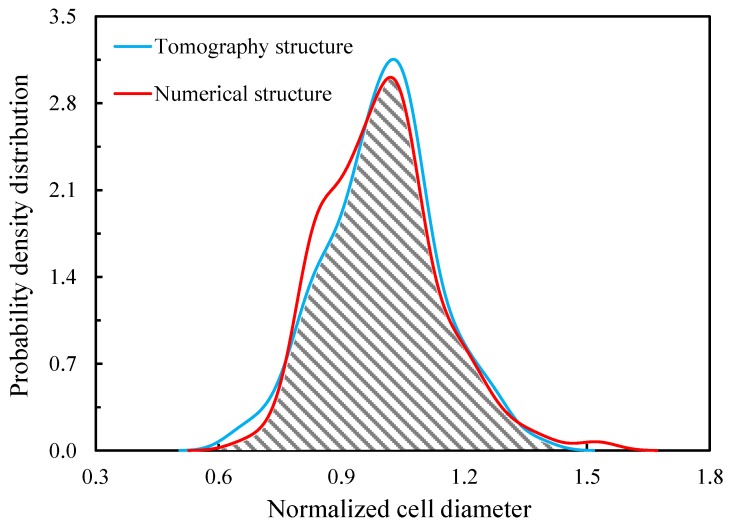
Normalized cell diameter distributions of the tomography structure and the numerical structure, as calculated by iMorph.

The cell size distribution of the tomography structure is plotted with the blue curve in Figure 5. Numerical closed-cell structures were generated with the aim of matching the coefficient of variation of the equivalent cell diameter distribution. The matching was realized by inputting the required quantity into the algorithm directly, rather than by means of an iterative process. The distribution of the normalized cell diameters of the numerical structure, i.e., the red curve in the figure, was very consistent with the blue curve. The two curves have a shared area fraction of 93.47%.

**Figure 6:**
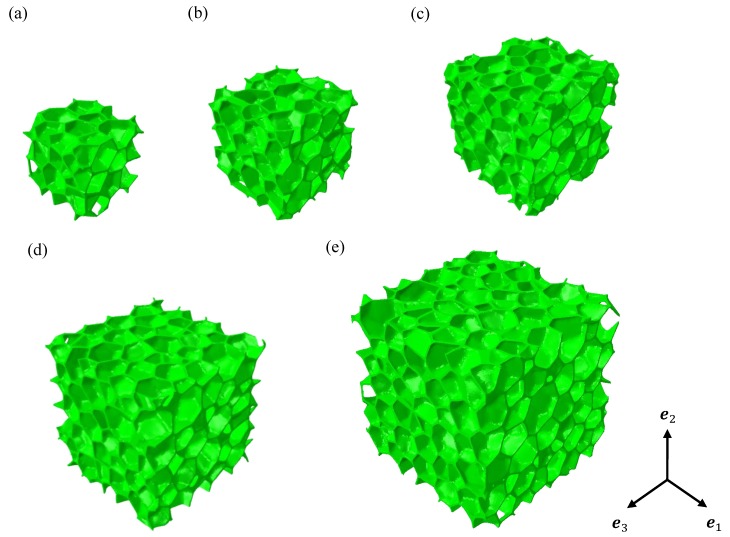
Illustration of the numerical irregular closed-cell model for (**a**) Set A; (**b**) Set B; (**c**) Set C; (**d**) Set D and (**e**) Set E.

In order to study the influence of the RVE parameters (the number of realizations and the RVE volume) [21,38,44,45], five sets of numerical closed-cell structures with different volumes were generated. Figure 6 and Table 1 show the illustration and the characterization of each set, respectively. The ratio of the number of cells to the volume of models was kept constant and was obtained by morphological analysis of the tomography structure.

## 3. Computational Homogenization

### 3.1. RVE Periodic Equilibrium State

On the fine scale, the studied foam is considered to be a heterogeneous medium composed of the bulk phase and the porous phase. The presence of the heterogeneities induces fluctuations in the local mechanical fields so that the displacement field, u, in a local point x∈Ω can be split into a mean part and a periodic fluctuation part $u^{\prime }\left(x\right)$

$$u\left(x\right)=\bar{\epsilon }\cdot x+u^{\prime }\left(x\right),$$

where $\bar{\epsilon }=\langle \epsilon \rangle$ is the average strain field $\epsilon =1/2\left(\nabla u{+}^{T}\nabla u\right)$ over the RVE. The state equilibrium equations for an RVE under the periodic boundary condition are governed by

$$\left\{\begin{array}{c} \mathrm{div}(\sigma (x))=0\\ \sigma \left(x\right)=C\left(x\right):\left(\bar{\epsilon }+\nabla_{s}u^{\prime }\left(x\right)\right)\\ u^{\prime }\;\mathrm{is}\;\mathrm{periodic}\;\mathrm{and}\;\sigma \cdot n\;\mathrm{is}\;\mathrm{anti}-\mathrm{periodic} \end{array}\right.,$$

where C(x) is the stiffness fourth order tensor of the foam, and the periodicity (resp. anti-peridicity) of a quantity means that this quantity takes the same (resp. opposite) value on opposite sides of the RVE boundary. The objective of the homogenization upscaling theory is to determine the effective behavior that links the average fields $\bar{\epsilon }$ and $\bar{\sigma }=\langle \sigma \rangle$. During the scale transition, the Hill–Mandel lemma postulates that the average value of the local energy is equal to the energy of the average fields [46]

$$\langle \sigma :\epsilon \rangle =\bar{\sigma }:\bar{\epsilon }.$$

The effective stiffness tensor $C^{\mathrm{hom}}$ satisfying $\bar{\sigma }=C^{\mathrm{hom}}:\bar{\epsilon }$ can be numerically derived in the framework of the finite element method using the VAMUCH method [34,47] or its Hill–Mandel equivalent formulations, as proposed in [21,25].

### 3.2. Finite Element Implementation

The generated structures were meshed with linear tetrahedron solid elements by the commercial package ICEM CFD 16.2 (ANSYS, Inc., Canonsburg, PA, USA) [48] and solved by the Hill’s lemma based periodic computational homogenization approach [21,25]. Hill’s lemma approach was recently implemented on open-cell models and has been verified as an efficient and accurate computational homogenization approach. It permits the full effective elastic stiffness matrix $\left[C^{\mathrm{hom}}\right]$ to be obtained with only one finite element computation and without imposing boundary macro-loadings [25], in contrast to the usual FEM homogenization approaches. According to [25], the homogenized elastic behavior $\left\{\bar{\sigma }\right\}=\left[C^{\mathrm{hom}}\right]\left\{\bar{\epsilon }\right\}$ can be written with the modified Voigt notation (any symmetric second order tensor a is represented by the vector $\left\{a\right\}{=}^{T}\left\{a_{11},a_{22},a_{33},\sqrt{2}a_{23},\sqrt{2}a_{13},\sqrt{2}a_{12}\right\}$ where $a_{ij}$ are its components in the basis $\left(e_{1},e_{2},e_{3}\right)$) as:

(2) $$\left\{\bar{\sigma }\right\}=\left(\left[\bar{C}\right]{+}^{T}\left[F\right]{\left[K\right]}^{-1}\left[F\right]\right)\left\{\bar{\epsilon }\right\}.$$

In Equation (2),

- $\left[\bar{C}\right]$ is the matrix representation of the Voigt bound of the elastic stiffness matrix of the studied RVE. In the case of a bulk-porous foam, it corresponds to $\left[\bar{C}\right]=\left(\rho^{\mathrm{hom}}/\rho^{b}\right)\left[C^{b}\right]$.
- The pseudo-force matrix [F] is obtained with the finite element assembly by computing
  $$\left[F\right]=\frac{1}{V}\int_{\Omega }{\left[B\right]}^{T}\left[C^{b}\right]dx,$$
  where the matrix [B] relates the fluctuation strain and displacement in the finite element discretization with $\left[\epsilon^{\prime }\right]=\left[B\right]\left[u^{\prime }\right]$.
- [K] is the FEM rigidity matrix that is inverted under the periodicity condition.

The detailed derivation of Hill’s lemma approach can be found in [25].

### 3.3. Mesh Sensitivity

**Figure 7:**
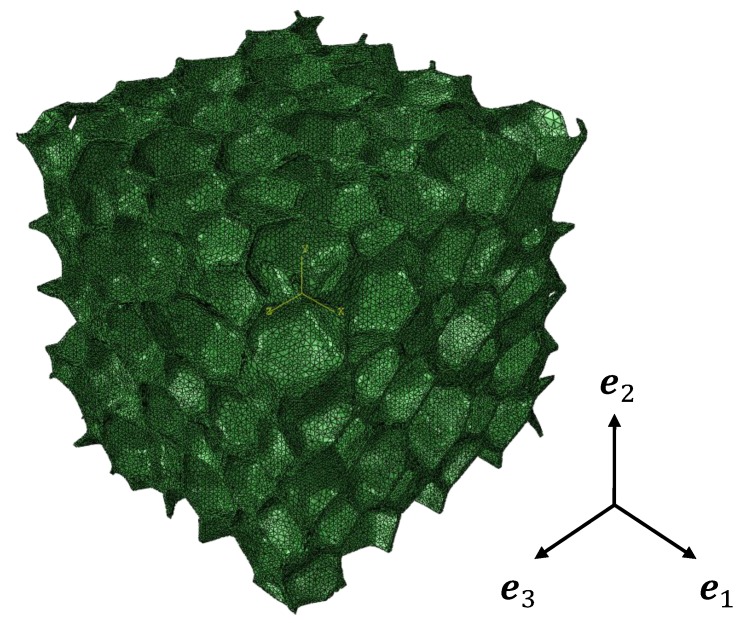
The periodic irregular closed-cell model with 821,087 elements.

**Figure 8:**
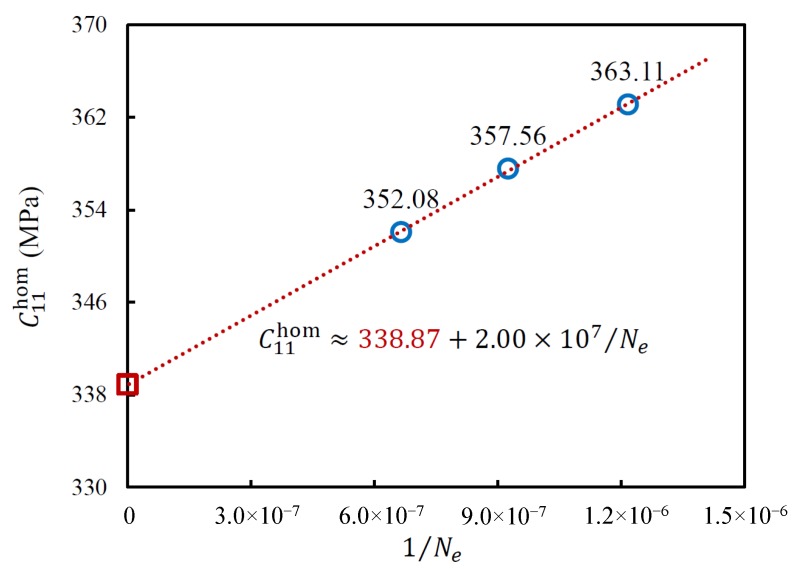
The relation between $C_{11}^{\mathrm{hom}}$ and the number of elements.

In order to reduce the mesh discretization error, the convergence approach was used in this study. The final result was obtained by converging the data with different mesh densities for each model. A linear equation was proposed [49,50] to present the relation between the component of the effective elastic stiffness matrix and the number of elements, which has the form:

(3) $$C_{ij}^{\mathrm{hom}}\approx C_{ij}^{0}+\frac{a}{N_{e}},\;\mathrm{with}\;i,j=1,2,3\ldots 6,$$

where $C_{ij}^{\mathrm{hom}}$ is one component of the effective elastic stiffness matrix, $C_{ij}^{0}$ stands for its convergence value, *a* means a constant, and $N_{e}$ denotes the number of elements. For example, a periodic irregular closed-cell structure (V=350×350×350 px${}^{3}$) was meshed with 821,087 elements (see Figure 7), 1,077,504 elements, and 1,389,842 elements, respectively. In Figure 8, the blue points are the calculated $C_{11}^{\mathrm{hom}}$ with different numbers of elements. The fitting line can be plotted with Equation (3). The intersection of the line and the *Y* axis, i.e., the red point, is the convergent result. The same convergence analysis was then performed for the other components of the effective stiffness matrix $\left[C^{\mathrm{hom}}\right]$.

Stochastic realizations were performed in order to take into account the microstructure irregularities of the studied closed-cell foams. Thus, the statistical mean stiffness matrix for $n_{r}$ realizations was computed with

$$\bar{C^{\mathrm{hom}}}=\frac{1}{n_{r}}\sum_{m=1}^{n_{r}}C_{m}^{\mathrm{hom}},$$

where *m* means the *m*th realization. For Set D, the overall effective elastic stiffness matrix after 5 realizations in the frame $\left(e_{1},e_{2},e_{3}\right)$ was

$$\left[\bar{C^{\mathrm{hom}}}\right]=\left[\begin{array}{cccccc} 340.51 & 153.47 & 152.84 & ∼0 & ∼0 & ∼0\\ & 342.91 & 153.08 & ∼0 & ∼0 & ∼0\\ & & 341.62 & ∼0 & ∼0 & ∼0\\ & Sym & & 188.02 & ∼0 & ∼0\\ & & & & 186.56 & ∼0\\ & & & & & 187.24 \end{array}\right]\left(\mathrm{MPa}\right).$$

A criterion $\zeta_{iso}$ [21,25,50] was then used to quantify the overall isotropy of the homogenized property. The isotropy criterion $\zeta_{iso}$ can be written as

(4) $$\zeta_{iso}=\frac{∥\bar{C^{\mathrm{hom}}}-P_{\mathrm{iso}}\left(\bar{C^{\mathrm{hom}}}\right)∥}{∥\bar{C^{\mathrm{hom}}}∥}\leq 2\%,$$

where the fourth order tensor norm is given as $∥T∥=\sqrt{T::T}$ and the projection operator $P_{\mathrm{iso}}\left(\bar{C^{\mathrm{hom}}}\right)$ over the isotropic basis is defined as

$$P_{\mathrm{iso}}\left(\bar{C^{\mathrm{hom}}}\right)=\frac{\bar{C^{\mathrm{hom}}}::J}{J::J}J+\frac{\bar{C^{\mathrm{hom}}}::K}{K::K}K$$

where J and K are the components of the isotropy basis of isotropic fourth order symmetric tensors. Using Equation (4) for Set D, the isotropy criterion of the overall effective matrix after 5 realizations was deduced as $\zeta_{iso}=0.28\%$. This indicates that the homogenized behavior can be assumed to be isotropic. Hence the effective elastic stiffness tensor can be equivalently written as

$$\bar{C^{\mathrm{hom}}}=3\bar{k^{\mathrm{hom}}}J+2\bar{\mu^{\mathrm{hom}}}K,$$

with an overall effective bulk modulus of $\bar{k^{\mathrm{hom}}}=215.98$ MPa and an overall effective shear modulus of $\bar{\mu^{\mathrm{hom}}}=93.89$ MPa. Due to the isotropic property, we focused on $k^{\mathrm{hom}}$ and $\mu^{\mathrm{hom}}$ in the current study.

## 4. Results and Discussion

### 4.1. Influence of RVE Parameters

**Figure 9:**
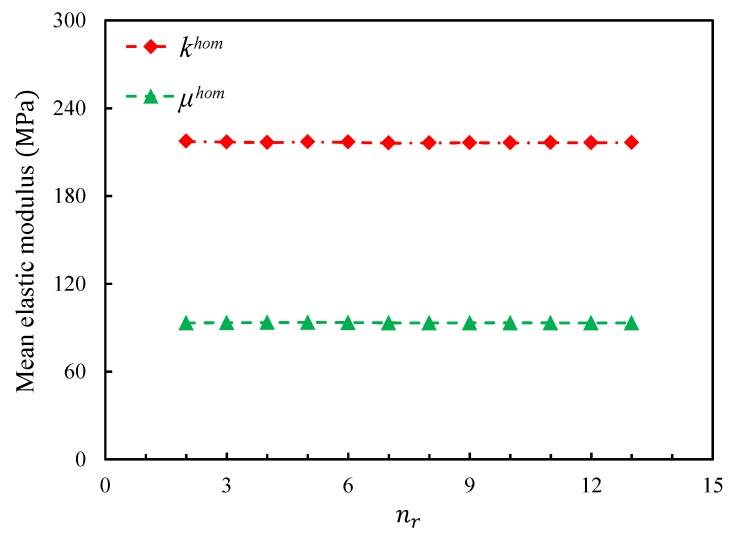
Evolutions of $\bar{k^{\mathrm{hom}}}$ and $\bar{\mu^{\mathrm{hom}}}$ as a function of the number of realizations for Set A.

The influence of the number of realizations $n_{r}$ on the effective elastic properties of the closed-cell foams was estimated first. Figure 9 shows the evolutions of the mean effective elastic moduli with an increase of the number of realizations for Set A. One can see that even for the smallest models, the variances of both moduli were negligible with very few realizations. The same tendency can be observed on the other sets.

**Figure 10:**
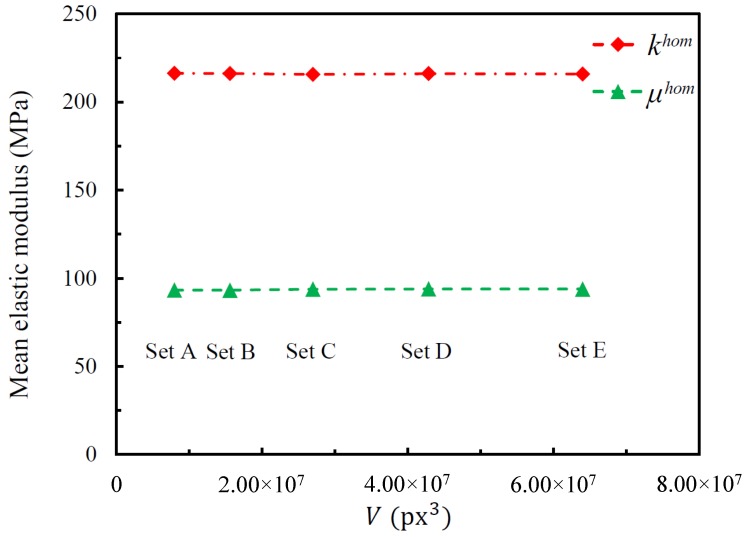
Evolution of $\bar{k^{\mathrm{hom}}}$ and $\bar{\mu^{\mathrm{hom}}}$ as a function of the RVE volume.

The evolutions of $\bar{k^{\mathrm{hom}}}$ and $\bar{\mu^{\mathrm{hom}}}$ with the increase of the volume of RVE are given in Figure 10. All data are the convergence values. In the figure, one can see for each effective elastic modulus, the differences among all 5 sets were very small. No bias caused by boundary effects was observed for these models. Since any set can represent this series of numerical models, the results of Set B were used in the following studies.

### 4.2. Influence of the Relative Density

**Figure 11:**
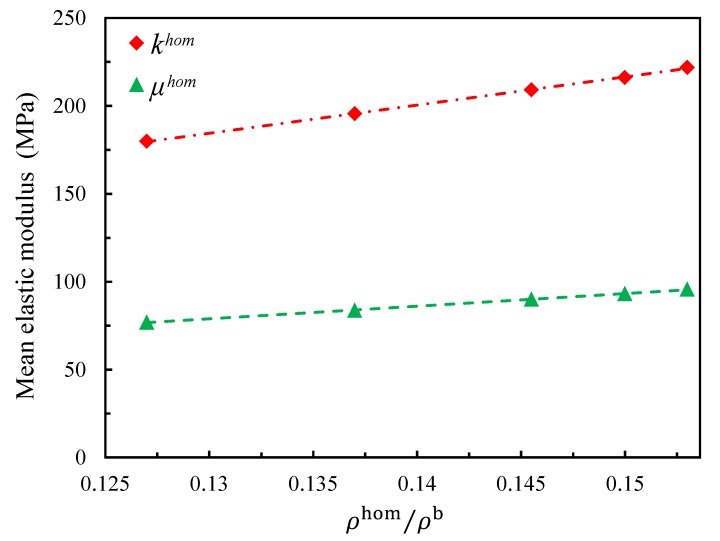
Effect of the relative density on the effective elastic moduli.

The influence of the relative density is detailed in this section. As said in Section 2.1, according to [36], the density of the PVC bulk varies from 1300 kg/m${}^{3}$ to 1580 kg/m${}^{3}$, which makes the corresponding relative density vary from 12.66% to 15.38% given that the foam density equals 200 kg/m${}^{3}$. Hence, different models were generated as in Set B except with different relative densities (12.7%, 13.7%, 14.6% and 15.3%). Since the numerical models were proven to be very stable in Section 4.1, for each relative density, four realizations were estimated and the mean results are used to show the influence. The evolutions of the effective elastic moduli with the relative density are presented in Figure 11. It is possible for there to be a non-linear evolution for a large relative density range; however, in this case, the evolution can be considered linear in the current small range.

### 4.3. Comparison with the Tomography Model

**Figure 12:**
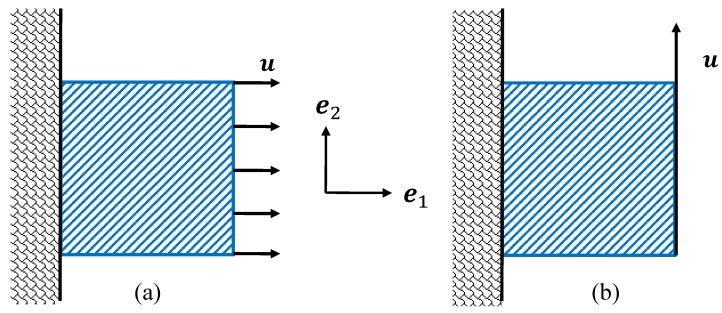
Illustration of (**a**) the tensile simulation and (**b**) the shear simulation from a 2D perspective.

**Figure 13:**
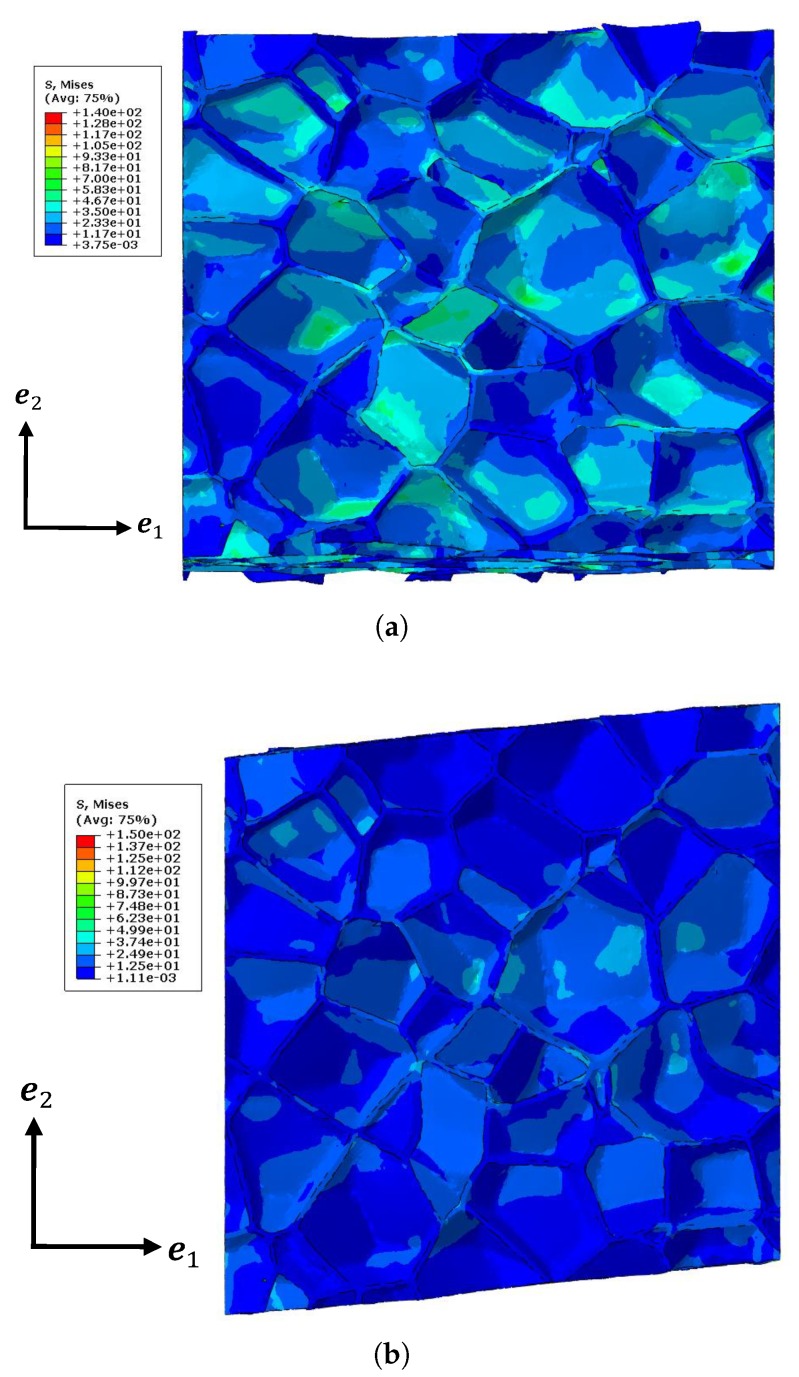
Deformations of the tomography model for (**a**) the tensile test and (**b**) the shear test with the *von Mises* equivalent stress map.

A comparison of the effective elastic properties between the tomography model (Figure 1b) and the homogenized model is detailed in this section.The relative density of the tomography model was set as 15%. The kinematic boundary conditions were imposed on the tomography model instead of the periodic boundary condition, since the structure is non-periodic. The tensile simulation and the shear simulation were carried out on models by applying the displacements on one surface along different directions while “clamping” the opposite surface along the corresponding directions (Figure 12 shows hla 2D perspective for simplicity). The tomography model was meshed with 654, 303, 969, 394, and 1,596,850 linear tetrahedron solid elements, respectively, and the convergence values were used for the comparison as well. The finite element simulations were realized by Abaqus. Figure 13 shows the deformations of the tomography (with 1,596,850 elements) after certain displacements for the tensile test and the shear test respectively with the *von Mises* equivalent stress map.

The overall macroscopic energy $\langle \psi \rangle =\langle \frac{1}{2}\sigma :\epsilon \rangle =\frac{1}{2}\bar{\sigma }:\bar{\epsilon }$ of the tomography model which was obtained by Abaqus simulation was compared to the homogenized derived behavior. Indeed, with Abaqus simulations, one can compute

$$\langle \psi \rangle =\frac{1}{\left|\Omega \right|}\sum_{e=1}^{N_{e}}\psi_{e}V_{e}$$

and

$$\langle \sigma \rangle =\frac{1}{\left|\Omega \right|}\sum_{e=1}^{N_{e}}\sigma_{e}V_{e},$$

where *e* means the element index, $\psi_{e}$ is the element strain energy, and $V_{e}$ stands for the volume of the element. On the other hand, the overall energy within the elastic RVE can be written for macroscopically isotropic media as

$$\psi^{\mathrm{hom}}=\frac{{\bar{\sigma }}_{m}^{2}}{2\bar{k^{\mathrm{hom}}}}+\frac{{\bar{\sigma }}_{eq}^{2}}{6\bar{\mu^{\mathrm{hom}}}}$$

where ${\bar{\sigma }}_{m}$ (resp. ${\bar{\sigma }}_{eq}$) corresponds to the macroscopic hydrostatic (resp. equivalent) stress obtained with Abaqus. Based on the previous computations, $\bar{k^{\mathrm{hom}}}$ and $\bar{\mu^{\mathrm{hom}}}$ for the homogenized model were determined to be 216.14 MPa and 93.21 MPa, respectively.

**Figure 14:**
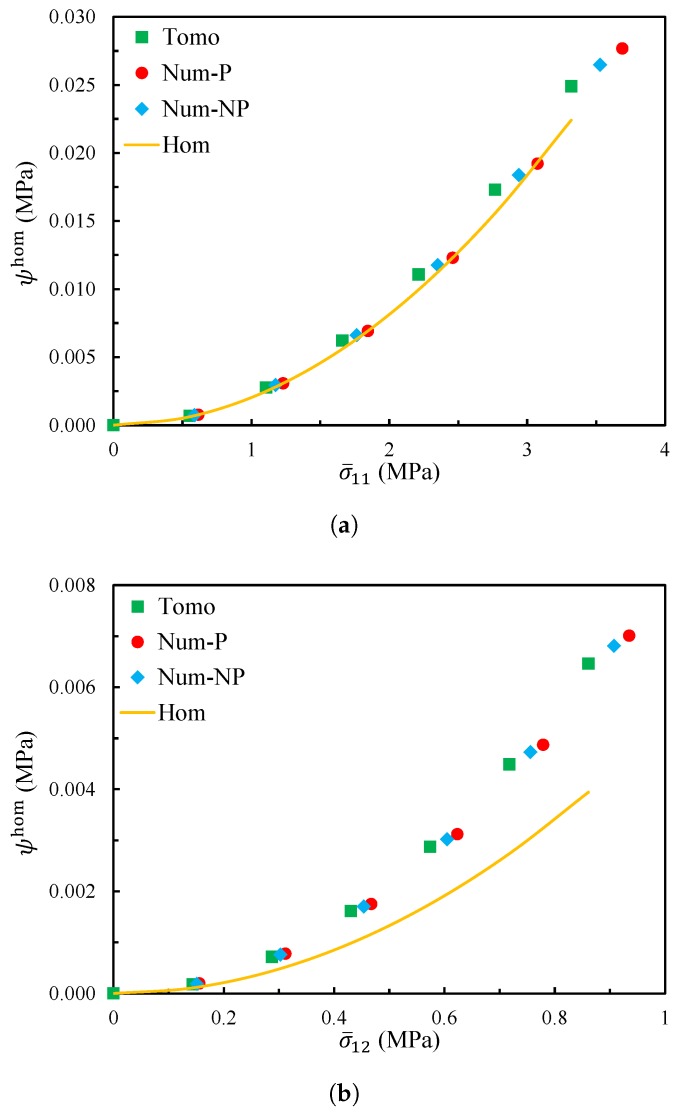
The evolution of the energy density as a function of (**a**) ${\bar{\sigma }}_{11}$ and (**b**) ${\bar{\sigma }}_{12}$ for each model.

For the uniaxial tension or shear without the periodic boundary condition, all other components of the average stress $\left\{\bar{\sigma }\right\}$ can be considered to be 0 compared to ${\bar{\sigma }}_{ij}$. For example, for the two cases in Figure 12, the average stresses were ${}^{T}\left\{{\bar{\sigma }}_{11},0,0,0,0,0\right\}$ and ${}^{T}\left\{0,0,0,0,0,\sqrt{2}{\bar{\sigma }}_{12}\right\}$. Hence, the evolutions of the energy density with the overall macroscopic stress component ${\bar{\sigma }}_{ij}$ were compared between the tomography model (a real non-periodic microstrucure) and the homogenized model (derived with the periodicity boundary condition assumptions) in Figure 14. In addition, two numerically generated models were tested under the kinematic boundary conditions, similarly to the tomography model. Both of them had the same size and statistical morphology description as the tomography model. One was generated with geometrically periodic boundaries, and the other was generated with geometrically non-periodic boundaries. Figure 14a shows the evolutions of the energy density with ${\bar{\sigma }}_{11}$, and Figure 14b presents those with ${\bar{\sigma }}_{12}$. In the figures, the points represent the simulation results under the kinematic boundary conditions for the real tomography model, named “Tomo”, and for the numerically generated models, named “Num-P” and “Num-NP”, respectively. The corresponding results of the homogenized model are shown with solid lines, named “Hom”.

From the figures, one can see that for both the tensile test and the shear test, the tomography model gave similar responses to the numerical models, with either geometrically periodic boundaries or non-periodic boundaries.

Thus, the numerically generated models did not bias the real tomography model. Furthermore, for the tensile test, the difference between the homogenized model and the tomography model was negligible, while for the shear test, the difference in energy density was much more significant. It seems that shear is more sensitive to the periodic boundary condition than tensile for the RVE with V=400×400×400 px${}^{3}$.

From the above, the tomography model is not large enough to have correct results without the periodic boundary condition.

### 4.4. Comparison with Experimental Results

**Figure 15:**
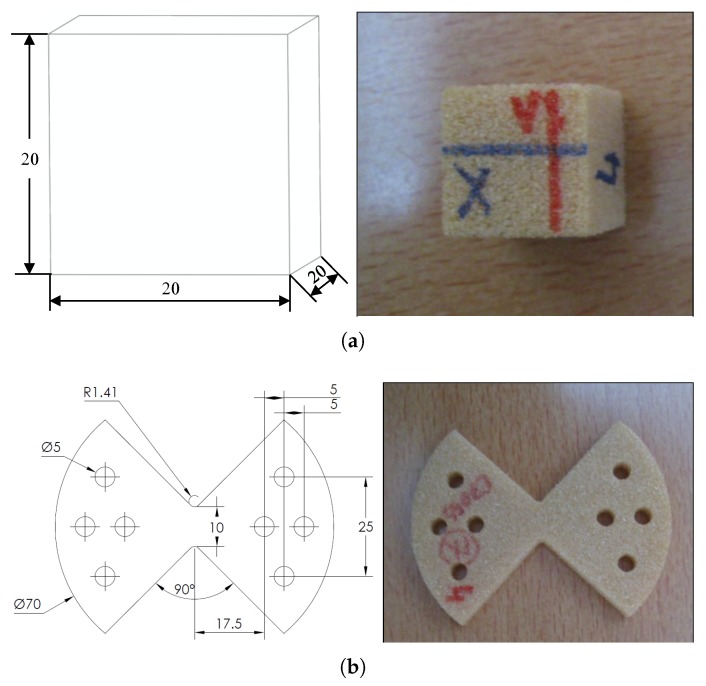
Illustration of (**a**) the cubic specimen for the compression test and (**b**) the “Batman”-shaped specimen for the shear test.

**Figure 16:**
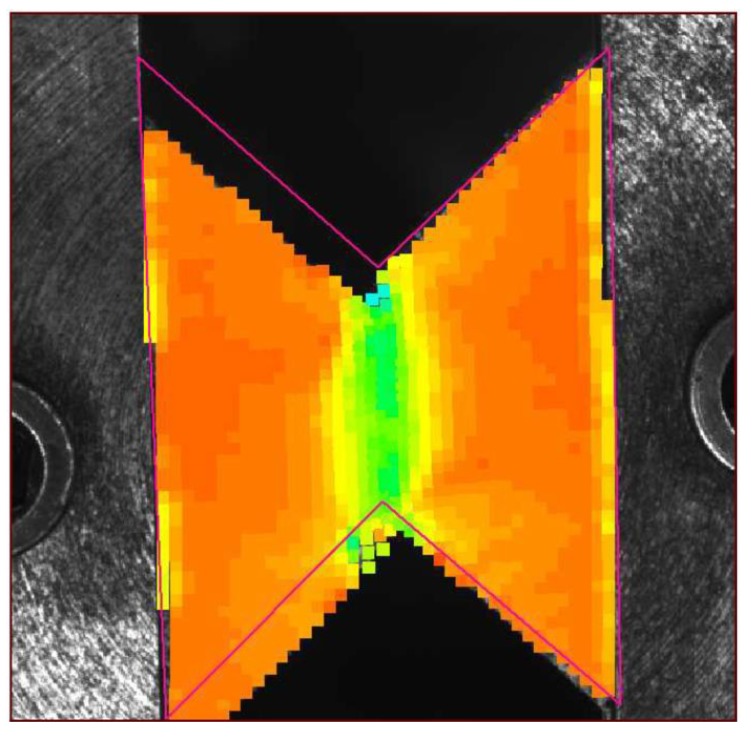
Illustration of the shear deformation of the “Batman”-shaped specimen.

Experiments were performed explicitly to obtain the macroscopic responses. The illustration of the specimens used for different tests is shown in Figure 15. The cubic specimen (20×20×20mm${}^{3}$) was prepared for the compression test, while the shear test is performed using the Arcan method [51,52,53] with the “Batman” type specimen, whose dimensions are marked in Figure 15b with a uniform thickness of 6 mm. It consisted of two half-moon shape flanges and was gripped in a loading frame with the load applied via the faces to avoid the potential problem of instability associated with edge loading and to obtain pure shear in the central part of the specimen. In the experiments, the local stain was measured with the digital image correlation technique [54]. Figure 16 is an example of the measurement of the strain field during the shear test using the software Icasoft [55].

**Figure 17:**
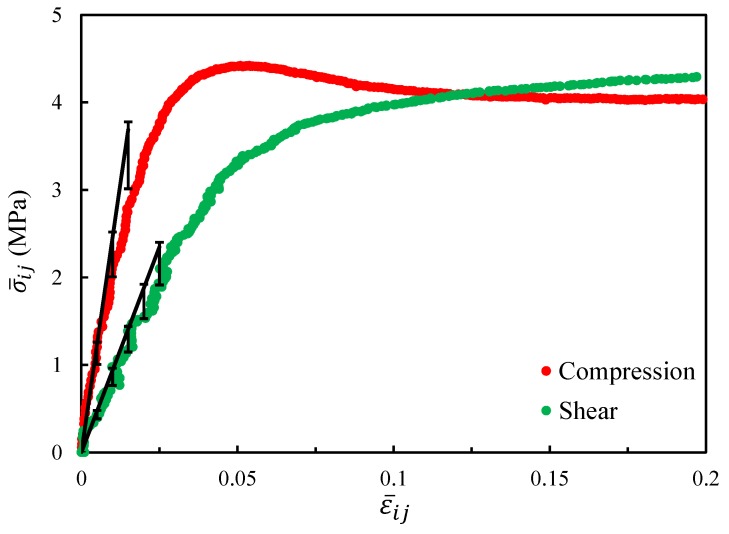
Stress–strain curves of the macroscopic compression and shear tests (the effective elastic moduli obtained by numerical simulations are shown with the black lines).

The macroscopic response curves are plotted in Figure 17. We focused on the elasticity of the responses in this study. Meanwhile, the corresponding effective elastic moduli of the homogenized model are shown by the black lines in the figure. Since the relative density ca not be determined accurately, the bars are used here to show the range of the effective elastic moduli caused by different relative densities in the range 12.66–15.38%. From the figure, one can see the homogenized results agreed well with the experimental results.

## 5. Conclusions

In this study, the effective elastic behavior of the irregular PVC closed-cell foams was evaluated. The main conclusions are as follows:

1. By analysing the tomography slices, the dispersion of cell size distribution and the anisotropy of the real irregular closed-cell foam were obtained. Using the approach based on Voronoi diagram, realistic irregular closed-cell foam structures were numerically generated with the morphological parameters.
2. The Hill’s lemma computational homogenization approach was used to predict the effective elastic behavior of the closed-cell foam models. The closed-cell foam models required only a small number of realizations to reach convergence. The influence of the relative density on the effective elastic moduli was studied. The approximately linear evolution was found in the small range. The energy density was investigated to compare the homogenized model with the tomography reconstruction model (without the periodic boundary condition). Two numerically generated models were also tested under the kinematic boundary conditions (similarly to the tomography model). One was generated with geometrically periodic boundaries and the other one was generated with geometrically non-periodic boundaries. Both of them had the same size and statistical morphology description as the tomography model. The results of these three models were very similar, while a deviation was observed with the homogenized model. This suggests that the numerically generated models did not bias the real tomography model. Furthermore, it suggests that the tomography sample was not large enough to obtain the correct results. The homogenized results were compared with the experimental results, and a satisfying agreement was achieved. Future investigations should be continued for larger samples to highlight the influence of size samples.

## Figures and Tables

**Table 1:** Characterizations of each set of closed-cell structures.

	Domain Dimensions (px${}^{3}$)	Number of Cells
Set A	200×200×200	30
Set B	250×250×250	58
Set C	300×300×300	100
Set D	350×350×350	159
Set E	400×400×400	238
